# RCPNEOPERU trial: a cluster randomized pilot trial to assess traditional neonatal resuscitation compared to partially virtual training in remote areas

**DOI:** 10.1016/j.jped.2025.04.008

**Published:** 2025-06-19

**Authors:** Carlos A. Delgado, Enrique Gómez Pomar, Pablo Velásquez, Víctor Sánchez, Roberto Shimabuku, Luis Huicho

**Affiliations:** a Universidad Nacional Mayor de San Marcos, Faculty of Medicine, Department of Pediatrics, Research Group in Neonatology (NEO), Lima, Peru; b Instituto Nacional de Salud del Niño, Neonatal Unit, Lima, Peru; c Universidad Peruana Cayetano Heredia, Centro de Investigación para el Desarrollo Integral y Sostenible (CIDIS) and Centro de Investigación en Salud Materna e Infantil (MAMAWAWA), Lima, Peru; d University of Kentucky, Department of Pediatrics, Lexington, KY, USA; e St Bernards Regional Medical Center, Jonesboro, AR, USA; f Instituto Nacional Materno Perinatal, Neonatal Unit, Lima, Peru; g Universidad Peruana Cayetano Heredia, Faculty of Medicine, Lima, Peru

**Keywords:** Infant, Newborn, Cardiopulmonary resuscitation, Staff development, Simulation training

## Abstract

**Objective:**

To assess the effects of a neonatal resuscitation training program using traditional training and partial distance learning.

**Method:**

Through an open cluster-randomized trial, the authors compared a traditional approach involving face-to-face theory and practice sessions using information and communication technology to offer theory and distance examination, followed by face-to-face practice. Twelve health facilities were allocated by blocked randomization. Comparisons were made adjusting for clustering in qualitative and quantitative data. The primary outcome was the percentage of infants with heart rates ≥100 per minute at the second minute after birth. The authors performed a cluster-level analysis for cluster randomized trials, simplifying the adjustment for individual- and cluster-level covariates.

**Results:**

The authors trained 403 health professionals in two arms in twelve facilities. After six months, the authors assessed 2180 birth deliveries, 966 newborns in the traditional training group (TT), and 1214 in the partial distance learning training group (pDL). The authors found no statistical evidence favoring any of the two trial arms (RR = 0.9859, CI 95 % = 0.9446; 1.0292, *p* = 0.4819).

**Conclusions:**

The authors found no statistical evidence favoring traditional or distance learning methods for neonatal resuscitation training. Further research could assess improved online platforms to enable sustainable virtual reality instructor/provider interaction for theory, practice and testing, addressed to health cadres of rural and remote areas.

## Introduction

Peru's neonatal mortality rate has decreased dramatically in the last two decades. However, gaps remain between the poorest and wealthiest quintiles and between urban and rural areas.^1^ Asphyxia accounts for >20 % of these deaths. Although skilled birth attendance coverage has increased,^2^ the management of newborns with asphyxia is suboptimal, due at least in part to the scarcity of adequately trained health personnel.

Neonatal resuscitation can help save lives and avoid long-lasting disabilities.^3^ However, training in neonatal resuscitation, particularly in remote rural areas, is challenging.^4^ There is no national program providing neonatal resuscitation training in Peru. Since 1999, the Social Security System (EsSALUD), which serves one-fifth of the population, has been running training for its staff.^5^ Also, sporadic training activities are mainly supported by non-governmental organizations, churches, or research groups.^6^^,^^7^

After reaching a historic low of 8.2 deaths per 1000 live births in 2020, Peru's neonatal mortality rate rebounded to 10 in 2023, effectively reversing the pre-pandemic decline.^8^^,^^9^ While 94.8 % of births now occur in hospitals, significant disparities persist, with healthcare coverage reaching 98.6 % in urban areas compared to 83.8 % in rural zones.^9^ These gaps are particularly evident in regions like Ayacucho and Cusco, where neonatal mortality rates remain above average.

Access to neonatal healthcare is crucial in remote and mountainous regions of Peru like Ayacucho and Cusco, where healthcare infrastructure is limited,^10^ and a significant proportion of newborn deaths occur at home or at peripheral health centers lacking resuscitation equipment and trained personnel.^11^

There is an urgent need to identify accessible and affordable alternative neonatal resuscitation training targeting remote areas. Therefore, the authors aimed to evaluate the effects of nontraditional distance training on neonatal resuscitation in healthcare facilities in Ayacucho and Cusco, Peru.

## Methods

### Trial

The authors conducted a pilot randomized cluster trial in twelve healthcare facilities. The number of necessary clusters was estimated using the minimum number of clusters needed for a matched design (i.e., six per arm).^12^ The authors opted for a cluster randomized trial design to minimize the risk of contamination between intervention and control groups. Each facility was considered a cluster, and thus it was randomly allocated to partially distance-learning methods (pDL) or to face-to-face traditional training (TT). The authors trained health providers in neonatal resuscitation. After six months, the authors assessed the effect of training. Detailed information on the study protocol has been published previously.^13^ The Flow process and pDL diagrams are shown in Supplementary File: Figures 1 s and 2s.

### Randomization

Healthcare facilities were matched by the proportion of non-medical professionals (nurses and obstetricians). After pairing for such characteristics, the authors allocated arms through blocked randomization using the R statistical software.

### Participants

The authors trained essential healthcare personnel teams of doctors, nurses, midwives, and nurse technicians. Five out of six instructors worked in a pediatric referral facility in Lima, Peru. Instructors in charge were two neonatal nurses and four neonatologists. Two neonatologists were mentor instructors trained by the Neonatal Resuscitation Program - American Academy of Pediatrics (NRP-AAP). Universidad Peruana Cayetano Heredia issued the certification for participants, for training purposes.

### Interventions

The intervention was a hybrid model combining online theoretical instruction with on-site simulation, focusing on neonatal resuscitation. The intervention pDL group received online theory and distance examination, complemented by face-to-face practice. The control group received TT, including face-to-face theory, exam, and practice in a one-day course led by neonatal resuscitation instructors. The pDL group received around 3 h of additional theory online and continuous access to this information. The pDL training methodology incorporated multimodal digital learning resources, including asynchronous access to structured online theoretical materials, immersive 360° virtual reality clinical simulations, and interactive case-based scenarios with real-time feedback mechanisms (Supplementary File: Figure 2s). There were several scheduled dates for every health facility, to maximize participant attendance.

The authors conducted the training courses from August to October 2017. Participants had access to printed books and inflatable mannequins in their health facilities. Participant facilities in the pDL group received six tablets and online access to the collection of neonatal resuscitation e-books from the NRP-AAP 7th edition, including the paperback neonatal resuscitation textbook in Spanish.

### Outcomes

The primary outcome was the proportion of infants with a heart rate of 100 beats per minute or more at the second minute of life (HR2M) by stethoscope auscultation. The authors selected HR2M because it is an adequate proxy for an effective response to neonatal resuscitation in the delivery room.^14^ HR2M was registered based on the APGAR score component and pulse oximeter data. In Ayacucho a fingertip handheld pulse oximeter model VE-H100B, Edan USA, San Diego, CA, USA was mainly used, while in Cusco it was the Model Number CX130 with Brand Name C30 plus, Seoul, Korea.

Secondary outcomes included: 1) Time to start positive pressure ventilation (PPV) after birth; 2) Time to achieve heart rate ≥ 100 per minute; 3) APGAR at 1st and 5th minutes; 4) Supplemental oxygen after 10 min; 5) Inspiratory oxygen fraction needed at 30 min after birth; 6) Mortality rate during the first 7 days; 7) Number of referrals during the first 7 days of life and; 8) Number of certified health professionals.

Detailed information is unavailable regarding facility infrastructure (equipment, neonatal resuscitation resources, or 24/7 coverage), clinical metrics (NICU transfers, live births/pre-training mortality rates), or additional staff profiles (neonatal experience, specific skills, or workflow practices). These parameters were not systematically recorded during the study.

### Implementation

The first phase of the project was carried out in 2017, starting with the randomization process to allocate arms for two types of training. The authors selected, paired, and randomized 12 health facilities in Ayacucho and Cusco.^13^ The second and final stage, six months after training, was aimed at retrieving information about birth attendance during 2018 by assessing newborn delivery using a registration form to compare outcomes (Delivery room observation sheet, Supplementary File: Figure 3s). The study included only vaginal deliveries to focus on spontaneous births, which are more prevalent in rural areas with limited access to surgical interventions.

Time after delivery was controlled using a stopwatch. Informed consent from the parents was a prerequisite for data registration. The authors coordinated with the health personnel who attend births and the heads of health facilities to complete the records. A monetary incentive was provided to the health facility for each card correctly completed by the staff. The authors also hired two field supervisors to visit each participating facility twice a month, ensuring the cards were completed correctly. The overall study was conducted between August 2017 and December 2018. There is no information about missing data and incomplete cards.

### Statistical analysis

The authors performed a cluster-level analysis for cluster randomized trials, simplifying adjustment for individual- and cluster-level covariates. The authors used STATA to handle continuous, binary, or rate outcomes, providing unadjusted or adjusted analyses with various effect measures.^15^ Previously, the authors ran a descriptive analysis of attendees on neonatal training courses by age and profession. A score ≥80 % was considered knowing or approved.

The authors calculated Z scores and percentiles for birth weight, gestational age (GA), and sex using the online Intergrowth data entry.^16^ The authors classified as “non-vulnerable” term newborns (37 to 42 weeks) who were born with an average weight (2,500 to 4,000 g) and were appropriate for GA (above the 10th percentile and below the 90th percentile). The authors considered “vulnerable” all newborns below those parameters. Physical examination measured GA using a Dubowitz variant named the Capurro method.^17^

The authors compared the means of quantitative and qualitative variables of the intervention and control groups through a student *t*-test and proportion comparisons, respectively. The authors used STATA/SE software version 18.0 (STATA Corp, College Station, TX, USA).^18^

### Ethics

The Institutional Ethics Committee of Universidad Peruana Cayetano Heredia (UPCH) approved the project (Registry N° 273-10-17). A written authorization was obtained from the regional health departments (DIRESAS) authorities involved in the study and the authorities of the health care facilities in Ayacucho and Cusco. The authors obtained written informed consent from the health personnel to participate in the neonatal resuscitation training activities, and from the mothers to collect information on delivery and newborns.

## Results

### Recruitment and staff training

The authors trained 403 health professionals in 12 health facilities, including 17 male and 103 female professionals exposed to the TT, while 45 male and 238 female professionals exposed to pDL. The authors did not collect data about the staff’s previous experience. The mean age was 40.9 years (SD 10.0) for TT and 44.0 years (SD 9.8) for pDL. Age difference was observed between approved and non-approved participants (TT: 40.9 ± 9.7 vs. pDL: 44.7 ± 9.8, *p* < 0.01), with TT-trained midwives and nurse technicians being younger than pDL-trained ones (midwives TT: 40.6 ± 6.7 vs. pDL: 46.1 ± 8.3, *p* < 0.01; nurse technicians TT: 37.9 ± 9.5 vs. pDL: 44.6 ± 9.4, *p* < 0.01).

Among 276 approved participants, 26 were physicians, 109 nurses, 72 midwives, and 69 technical professionals. Overall, 403 health professionals participated at some point in the training process, while some attended only part of the training due to schedule conflicts. Of 403 enrolled, 352 (87.3 %) completed training across 12 sites, with 276 (78.4 %) achieving passing scores (>11/20).

### Assessment of staff performance in newborn care vaginal delivery

Data were collected prospectively throughout 2018 from the birth registry logs of each health center. Around six months after training, the authors registered data from 2180 deliveries using a printed ad-hoc registration form. The average birth weight of newborns in the TT group was 3244 g (SD 381), compared to 3257 g (SD 411) in the pDL group (*p* = 0.9339). The GA was similar in both training groups (*p* = 0.8482). Table 1 shows birthweight and GA data for 2180 neonatal participants by training group and newborn vulnerability.

**Table 1:** Birthweight, gestational age and Apgar comparison for 2,180 newborns. Distributed by neonatal wellbeing and training group.

Variables	Non-vulnerable newborn				*P* value	Vulnerable newborn				*P* value
	Traditional		pDL			Traditional		pDL		
Region and sex	***n***	***%***	***n***	***%***		***n***	***%***	***n***	***%***	
Ayacucho: Female	179	48.12	268	50.95	0.6288	18	47.37	16	39.02	0.4542
Male	193	51.88	258	49.05		20	52.63	25	60.98	
Cusco: Female	280	55.23	310	50.99	0.3248	23	46.94	21	53.85	0.5197
Male	227	44.77	298	49.01		26	53.06	18	46.15	
Heart rate 2nd min >100^b^	***n***	***%***	***n***	***%***		***n***	***%***	***n***	***%***	
Ayacucho region	370	99.46	499	94.87	0.3297	38	100	39	95.12	0.3442
Cusco region	498	98.22	604	99.34	0.3442	49	100	38	97.44	0.3442
	***n***	$\bar{x}$***[CI95 %]***	***n***	$\bar{x}$***[CI95 %]***		***n***	$\bar{x}$***[CI95 %]***	***n***	$\bar{x}$***[CI95 %]***	
Heart rate 2nd min >100^c^	383	142 [121; 164]	687	140 [126; 154]	0.8297	38	140 [111; 169]	55	139 [121; 156]	0.9176
Birthweight (k) Female	459	3.247 [3.146; 3.348]	578	3.253 [3.162; 3.344]	0.9073	41	2.600 [2.415; 2.785]	37	2.535 [2.350; 2.719]	0.5362
Male	420	3.362 [3.262; 3.462]	556	3.362 [3.268; 3.456]	0.9960	46	2.710 [2.438; 2.983]	43	2.589 [2.327; 2.851]	0.4271
Gestational age (weeks)	***n***	$\bar{x}$***(± SD)***	***n***	$\bar{x}$***(± SD)***		***n***	$\bar{x}$***(± SD)***	***n***	$\bar{x}$***(± SD)***	
Female	459	39.2 (± 2.22)	578	39.1 (± 2.24)	0.3970	41	38.0 (± 3.28)	37	37.8 (± 3.10)	0.7606
Male	420	39.3 (± 2.37)	556	39.1 (± 2.56)	0.4707	46	37.5 (± 3.78)	43	37.3 (± 3.58)	0.7831
Apgar 1-minute score	879	8.5 (± 8.29)	1134	8.3 (± 8.74)	0.5731	87	8.4 (± 2.06)	80	8.0 (± 1.95)	0.2796
Apgar 5-minute score	879	9.4 (± 2.52)	1134	9.2 (± 2.65)	0.0405	87	9.4 (± 0.81)	80	9.0 (± 0.80)	0.0381

Traditional, traditional training; pDL, partially distance learning.

*P* value, comparison adjusted for clustering in Qualitative data (Ztest, proportions), and Quantitative data (cltest and clchi2 commands using STATA).

Vulnerable newborns, are preterm newborns, low birthweight, or small birthweight for their gestational age. Non-vulnerable newborns did not accomplish the vulnerable newborn classification.

**Figure 1:**
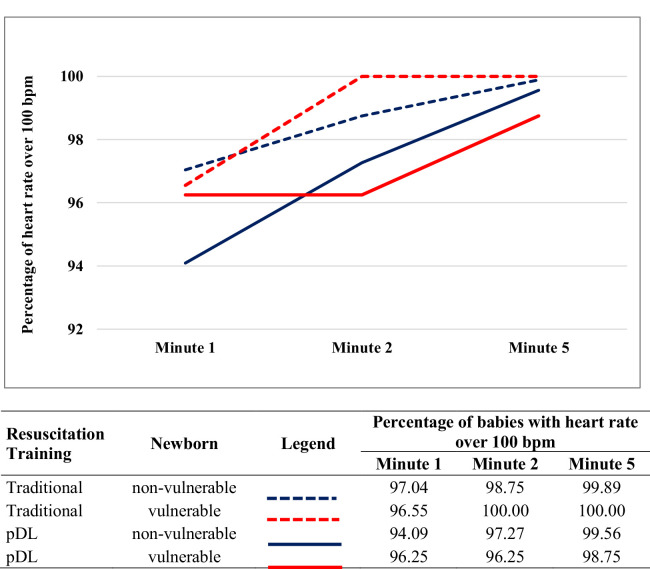
Percentage of babies achieving a heart rate over 100 beats per minute at the first, second, and fifth minute of their life. Traditional, traditional training; pDL, partially distance learning.

Table 2 shows the rate at two minutes of life in the TT and pDL groups. Overall, 2180 measurements from 12 health facilities based on pulse rate for the APGAR score (method 1) and 1163 measurements based on pulse oximetry (method 2) were available in 11 health facilities. There was no major difference in the primary outcome between both training groups (Figure 1).

**Table 2:** Heart rate at two minutes of life in 2,180 newborns.

Variables	Training group (Trial Arm)				*P* value
	*n*	Traditional	*n*	pDL	
Proportion of Heart rate ≥100 bpm at 2 min, measured by Apgar score					
Ayacucho region	410	99.5 [98.8; 100]	567	94.9 [93.1; 96.7]	0.3185
Cusco region	556	98.4 [97.3; 99.4]	647	99.2 [98.6; 99.9]	0.4390
Ayacucho and Cusco regions	966	98.9 [98.2; 99.5]	1214	97.2 [96.3; 98.1]	0.4547
Proportion of heart rate ≥ 100 bpm at 2 min, measured by Pulse Oximetry					
Ayacucho region	14	100 [100;100]	361	79.8 [93.8; 97.9]	0.8864
Cusco region	407	99.8 [99.3; 100]	381	100 [100;100]	0.5518
Ayacucho and Cusco regions	421	99.8 [99.3; 100]	742	98.0 [96.9; 98.9]	0.4744
Mean heart rate in bpm at 2 min of life, measured by pulse oximetry					
Ayacucho region	14	148 [41; 254]	361	143 [119; 167]	0.6785
Cusco region	407	142 [98; 186]	381	137 [91; 183]	0.7664
Ayacucho and Cusco regions	421	142 [120; 164]	742	140 [126; 154]	0.8406

Traditional, traditional training; pDL, partially distance learning; bpm, beats per minute.

*p* value, Comparison group adjusted for clustering.

(cltest and clchi2 commands using STATA).

Number [;] = Percentage [95 % confidence interval].

Table 3 presents the proportion estimates for achieving a heart rate over 100 bpm two minutes after birth (HR2M). The bivariate analysis showed no significant differences in the target heart rate when assessed by type of training (pDL or TT), region, prematurity, adequacy for GA, low birth weight, vulnerable newborn, sex, and weight Z-Score. However, the multivariate analysis showed a significant impact of GA on achieving the target heart rate (*p* = 0.0298). The multivariate analysis revealed that a lower weight Z-score was significantly associated with lower rates of target heart rate.

**Table 3:** Proportion estimates for achieving heart rate over 100 bpm in two minutes: bivariate and multivariate analysis.

Variable	Bivariate analysis				Multivariate analysis				Model
	Estimate	[95 % Conf. Interval]	df	*P*>|t|	Estimate	[95 % Conf. Interval]	df	*P*>|t|	
hr2m >100	0.9859	[0.9446, 1.0292]	10	0.4819	0.9859	[0.9446, 1.0292]	10	0.4819	m0
Branch	1.0028	[0.9597, 1.0477]	9	0.8889	1.0028	[0.9597, 1.0477]	9	0.8889	m1
Region	0.9859	[0.9447, 1.0289]	9	0.4725	1.0029	[0.9597, 1.0477]	8	0.8773	m2
know	0.9859	[0.9444, 1.0292]	9	0.4745	0.9989	[0.9595, 1.0398]	7	0.9495	m3
PREM	0.9860	[0.9443, 1.0295]	10	0.4845	0.9989	[0.9596, 1.0398]	7	0.9497	m4
AGA	0.9859	[0.9442, 1.0294]	10	0.4814	0.9989	[0.9596, 1.0398]	7	0.9499	m5
LBW	0.9857	[0.9441, 1.0291]	10	0.4745	0.9989	[0.9595, 1.0398]	7	0.9490	m6
SVN	0.9861	[0.9443, 1.0296]	10	0.4860	0.9989	[0.9597, 1.0396]	7	0.9487	m7
sex	0.9859	[0.9442, 1.0293]	10	0.4803	0.9989	[0.9597, 1.0369]	7	0.9485	m8
Weight Z-Score	0.9862	[0.9446, 1.0296]	10	0.4891	0.9748	[0.9246, 1.0277]	7	0.0291	m9
Gestational Age	0.9860	[0.9439, 1.0299]	10	0.4884	0.9749	[0.9241, 1.0285]	7	0.0298	m10

m0, hr2m > 100; m1, hr2m > 100, Branch; m2, hr2m > 100, Branch, Region; m3, hr2m > 100, Branch, Region, know; m4, hr2m > 100, Branch, Region, know, PREM; m5, hr2m > 100, Branch, Region, know, PREM, AGA; m6, hr2m > 100, Branch, Region, now, PREM, AGA, LBW; m7, hr2m > 100, Branch, Region, know, PREM, AGA, LBW, SVN; m8, hr2m >100, Branch, Region, know, PREM, AGA, LBW, SVN, sex; m9, hr2m > 100, Branch, Region, know, PREM, AGA, LBW, SVN, sex, Weight Z-Score; m10, hr2m > 100, Branch, Region, know, PREM, AGA, LBW, SVN, sex, Weight Z-Score, Gestational Age.

Branch, traditional training, partially distant learning; Region, ayacucho, Cusco; know, trainees with approved score (yes/no); PREM, preterm; AGA, adequate for gestational age; LBW, low birthweight; SVN, small vulnerable newborn; Sex, male/female; weight Z-score, intergrowth 21st Z-score; Gestational age, measured in weeks by physical exam (Capurro method).

Premature newborns represented 2.7 % of the participants. Almost 93 % of them were late preterm, equal to or above 34 weeks, and they had similar requirements for resuscitation as most of the evaluated newborns.

### Secondary outcomes

Early respiratory support using PPV was required in five TT-group infants vs. three pDL-group infants (all >2.5 kg except one pDL-group [birthweight 640 g, 23 weeks]). Cardiac rates above 100 bpm at the first minute of life favored the TT group (97 % with HR >100 bpm vs. 94 % in pDL, *p* < 0.01), while initial APGAR scores ≥ 7 in the first minute, were paradoxically higher in the pDL group (97.6 % vs. 95.4 %, *p* < 0.01). By the 5th minute, near-universal stabilization was achieved in both groups (TT:99.9 % vs pDL:99.8 % with APGAR ≥ 7, *p* = 0.436), demonstrating an equivalent outcome despite early differences. The single mortality (640 g pDL infant) represents an extreme preterm outcome unrelated to the intervention type. Four babies were referred to a high-level facility before day 7 of life, two of them born in Ayacucho and two in Cusco.

## Discussion

The authors found no statistical evidence favoring traditional or distance learning methods. Traditional NRP learning methods can be more challenging in remote rural areas. Therefore, distance learning can be a helpful alternative. Although different training strategies may be relevant under simulated scenarios, any training demands competent performance in real life. Greater access at a lower cost may be an additional advantage for distance learning strategies.

### Strengths

The main strength of this study is the use of a cluster-randomized study design, which offers robustness by minimizing contamination effects among participants within the same cluster. It ensures that interventions are implemented uniformly across entire facilities, reducing bias and enhancing the reliability of results. The authors chose as the primary outcome an objective measure of heart rate, which is a good indicator of adequate resuscitation.^14^^,^^19^ Heart rate is also routinely used in the delivery room and thus can be easily implemented within real-world healthcare contexts.

### Limitations

First, the authors calculated a sample size of 4000 deliveries to detect a difference of up to 4 % in the primary outcome.^13^ Although the authors collected data from 2180 participants and detected around 1 % difference in the primary outcome between training groups, this slight difference was not found to be statistically significant. Despite falling short of reaching the target sample size, the lower-than-expected variability within the sample reached might have contributed to a more precise estimate of the effect size.

According to a Peruvian birth reporting system, 5,710 children were born in the regional facilities during the study period.^20^ Vaginal births accounted for 68 % of the total (3,888), and the authors observed more than half of those births (56 % = 2,180).

Second, the authors could not conduct a baseline study, as it was assumed that the blocked randomization process balanced the distribution of conglomerates. Participants in both training groups are relatively homogeneous regarding the type of professionals and qualifications during training. However, there was a significant age difference between those who passed and those who did not, with midwives and nurse technicians in the TT group being nearly six years younger on average. This may reflect greater professional experience in the PDL group. In addition, only 78.4 % of participants completed theoretical and practical evaluations, potentially affecting the generalizability of the findings.

The 78.4 % approval rate (score > 11/20) likely reflects several contextual challenges as participants' advanced mean age (>40 years) is potentially associated with reduced digital literacy; high staff turnover in rural facilities as professionals migrate toward urban centers; and absence of baseline competency assessments. While the authors did not evaluate learning curves or clinical performance impact, these results highlight structural training barriers in remote settings. The passing threshold (>55 % of the total score) aligned with national standards but fell below the 75 % benchmark common in better-resourced settings.^21^ This disparity underscores the need for context-adapted evaluation systems that maintain minimum competency requirements without disregarding local realities.

Clustered data analysis has some limitations.^15^ Using a low number of clusters reduces statistical power, increases sensitivity to outliers, and presents challenges in adjusting for confounders.^22^ However, the analysis remains useful for exploratory analysis and hypothesis generation.

The authors acknowledge that using a 2-minute APGAR score deviates from standard practice, which typically measures APGAR at 1 and 5 min. The present study focused on the 2-minute heart rate as a specific indicator of early resuscitation effectiveness. When available, the heart rate was measured using multiple methods, including stethoscope auscultation, palpation, and pulse oximetry. While oximeters were available in most centers, they were not consistently used due to staffing issues and delays in equipment readiness during resuscitation events. In addition, while prior studies^23^ suggest that sensor application timing influences signal quality, the protocol ensured pulse oximetry sensors were systematically placed within the first minute of life in all cases. Although specific initial conditions (e.g., moisture, perfusion) were not recorded, this standardized approach minimized variability in monitoring initiation. Thus, the data reflect continuous measurements from the immediate postnatal period, optimizing the capture of critical heart rates during neonatal transition. Each method for heart rate measurement has its own variability,^24^ and the authors did not grant access to the more accurate electrocardiogram. Also, the authors did not evaluate using beta-blockers or any other cardiovascular treatment in mothers.

The lower mortality rate observed in the present study may be due to several factors. The present study’s sites were healthcare facilities with whole staff to handle neonatal resuscitation, as opposed to home births or under-resourced settings with higher mortality rates. Additionally, the population included in this study may not be fully representative of the broader national population, particularly those in more remote areas.

Finally, financial compensation for data collection may have introduced some additional bias. To mitigate this risk, the authors implemented several verification procedures. Independent data monitors audited data collection forms and regularly visited each participating center to verify records against hospital logs.

### Generalizability

The authors conducted this pilot study in Ayacucho and Cusco, two resource-limited regions in Peru's central and southern highlands with high neonatal mortality rates. The positive feedback from participants regarding virtual learning methods, such as virtual reality video simulation, suggests the potential for these approaches to be applicable across diverse settings. Moreover, the growing availability and utilization of virtual learning platforms, especially in resource-limited healthcare facilities, highlights the relevance of the present findings beyond Peru's borders. Given the universal need for neonatal resuscitation training and the increasing accessibility of virtual learning tools, the present study underscores the importance of exploring non-traditional training strategies on a broader scale, potentially benefiting healthcare providers in other settings.

The 0.4 % rate of PPV requirement in this study contrasts with the 4.4 % prevalence reported in comparable high-neonatal-mortality settings, such as the EN-BIRTH study.^25^ This marked difference primarily reflects the population's mature GA profile (median 39 weeks), given the well-documented inverse relationship between GA and PPV need (2.6 % in latepreterm vs 85 % in extremely preterm infants.^26^ While these findings suggest effective stabilization of term and near-term neonates, they underscore the critical need for GA-stratified resuscitation benchmarks in diverse resource settings, particularly given the non-linear relationship between birth weight and respiratory transition.

The present study's few advanced resuscitative interventions limit the ability to draw definitive conclusions about the competence gained through different training methods. The authors also found that the 5-minute APGAR scores were statistically higher in the traditional training group (9.4 vs 9.2), although small. Of note, there is no widespread use of laryngeal masks in neonatal resuscitation anywhere.^27^^,^^28^

Over the past years, virtual learning has become more available and widely used. Video simulation was evaluated in a pilot study in Peru with 20 participants who completed a survey after a video simulation of a pediatric patient in respiratory distress. Participants reported positive feedback, especially on reviewing medication dosing and the skills required for performing chest compressions.^29^ In 2021, Velasquez-Velasquez et al.,^30^ did a systematic review of virtual training compared to standard cardiopulmonary resuscitation for neonates and infants. They found that both training approaches are associated with an adequate acquisition of basic concepts. In contrast, the traditional approach is associated with a better performance in acquiring technical skills. Another study found that even one hour of simulation may improve medical students' performance in the initial steps of the neonatal resuscitation algorithm and mask ventilation skills.^31^

Previous studies have shown a decline in resuscitation skills over time,^4^^,^^32^ emphasizing the importance of regular in-situ training and simulation activities. Training in neonatal resuscitation in an Andean country with diverse geography and difficult access, like Peru, requires innovative strategies. Traditional training is still valuable, but affordability constraints and continuous updates may limit the training of health personnel who attend births in remote health facilities. The study was performed some years before the last neonatal resuscitation guideline in 2020.^3^ Regardless, those new guidelines did not change the objective. It is still necessary to assess other benefits, such as instructor telepresence for distance practice training, theory, and distance examination. Based on current evidence, a model mixing virtual theory and in-person skills review can be implemented, particularly in remote areas.

In conclusion, the authors found no statistical evidence favoring traditional or distance learning methods for neonatal resuscitation training. Traditional face-to-face training in rural settings presents several challenges, including continuous updates, availability, and affordability problems that limit health personnel's efficient training. It is necessary to assess alternative neonatal resuscitation training approaches further, with improved platforms allowing the virtual availability of instructors during distance theory and practice courses and virtual examination activities.

## Authors’ contributions

Carlos A. Delgado: Conceptualization, methodology, funding acquisition, project administration, investigation, data curation, formal analysis, writing–original draft, writing–review & editing.

Enrique Gómez Pomar: Investigation, formal analysis, writing–review & editing.

Pablo Velásquez: Investigation, writing–review & editing.

Víctor Sánchez: Investigation, writing–review & Editing.

Roberto Shimabuku: Methodology, writing–original draft, writing–review & editing, supervision.

Luis Huicho: Methodology, funding acquisition, project administration, investigation, formal analysis, writing–review & editing, supervision.

## Conflicts of interest

The authors declare no conflicts of interest.

## Appendix Supplementary materials

Image, application 1

## Footnotes

Supplementary material associated with this article can be found in the online version at doi:10.1016/j.jped.2025.04.008.
