# Supplementary material for: RCPNEOPERU trial: a cluster randomized pilot trial to assess traditional neonatal resuscitation compared to partially virtual training in remote areas
Source: J Pediatr (Rio J). 2025 Jun 19;101(4):520–8. doi: 10.1016/j.jped.2025.04.008 (PMC12276612; doi:10.1016/j.jped.2025.04.008)
Supplement: Supplementary file 1 [file mmc1.docx]

# **JPED-D-25-00071_Supplementary Material**

**Supplementary File**: Figure 1s

**Figure 1s** Flow diagram for cluster enrolment, allocation, and analysis.

Eligibility assessment (n = 18 clusters)

Randomised (n = 12 clusters)

Excluded (n = 6 clusters)

Not meeting inclusion criteria (n = 4)

Ayacucho: Puquio, Coracora,

Cusco: Santo Tomas, Espinar

Declined to participate (n = 0)

Other reasons: VRAEM (n = 2)

Ayacucho: San Francisco; Cusco: Kimbiri

Allocated to intervention (n = 6 clusters)

Received allocated intervention (n = 6)

Did not received intervention (n = 0)

Allocated to intervention (n = 6 clusters)

Received allocated intervention (n = 6)

Did not received intervention (n = 0)

Lost to follow-up (n = 0 clusters)

Discontinued intervention (n = 0)

Lost to follow-up (n = 0 clusters)

Discontinued intervention (n = 0)

Analyzed (n = 6 clusters)

Excluded from analysis (n = 0)

Analyzed (n = 6 clusters)

Excluded from analysis (n = 0)

**Analysis Follow-up Allocation Enrolment**

**VRAEM**, Valley of the Apurímac, Ene and Mantaro rivers' (It is one of the major areas of coca production in Peru).

**Supplementary File**: Figure 2s

**Figure 2s** Visual comparison between Distance-Learning (pDL) and Traditional-Training (TT).


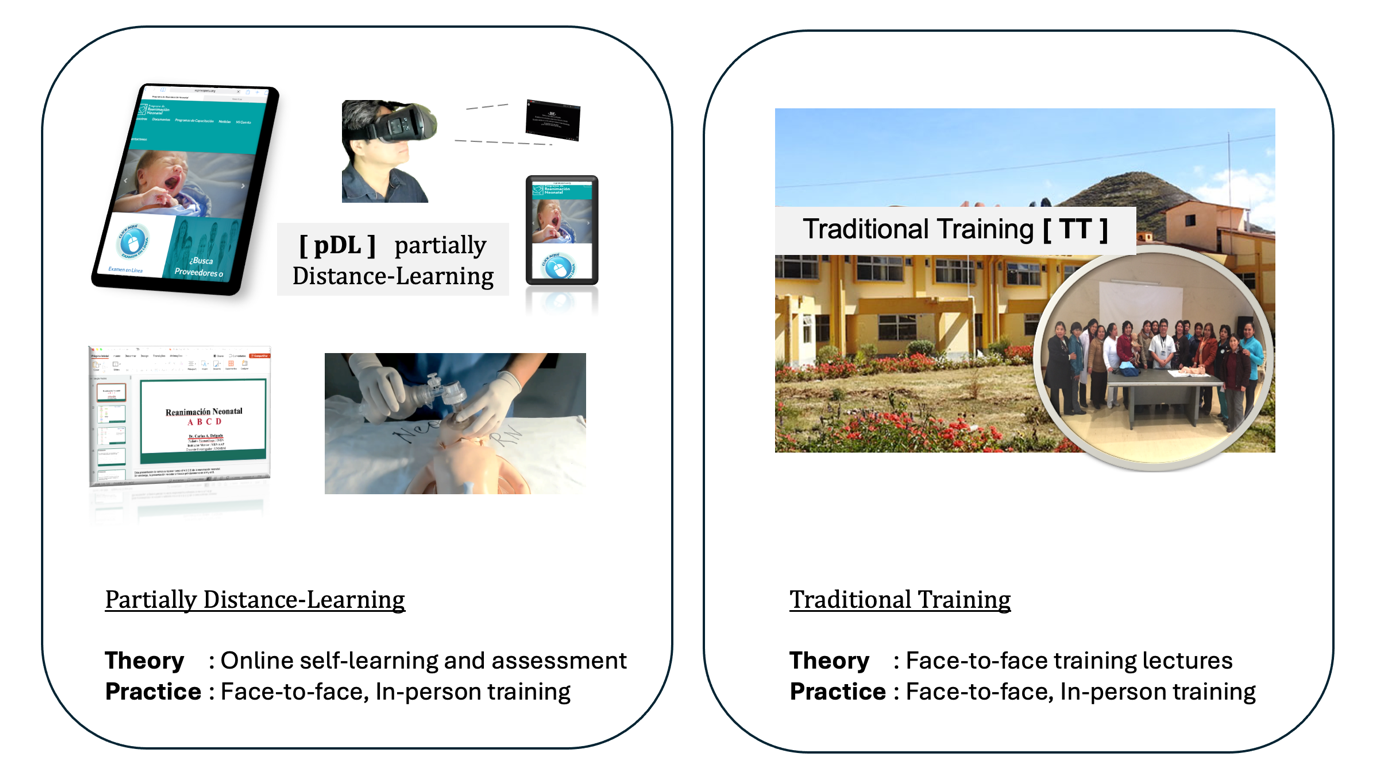


**Supplementary File**: Figure 3s

**
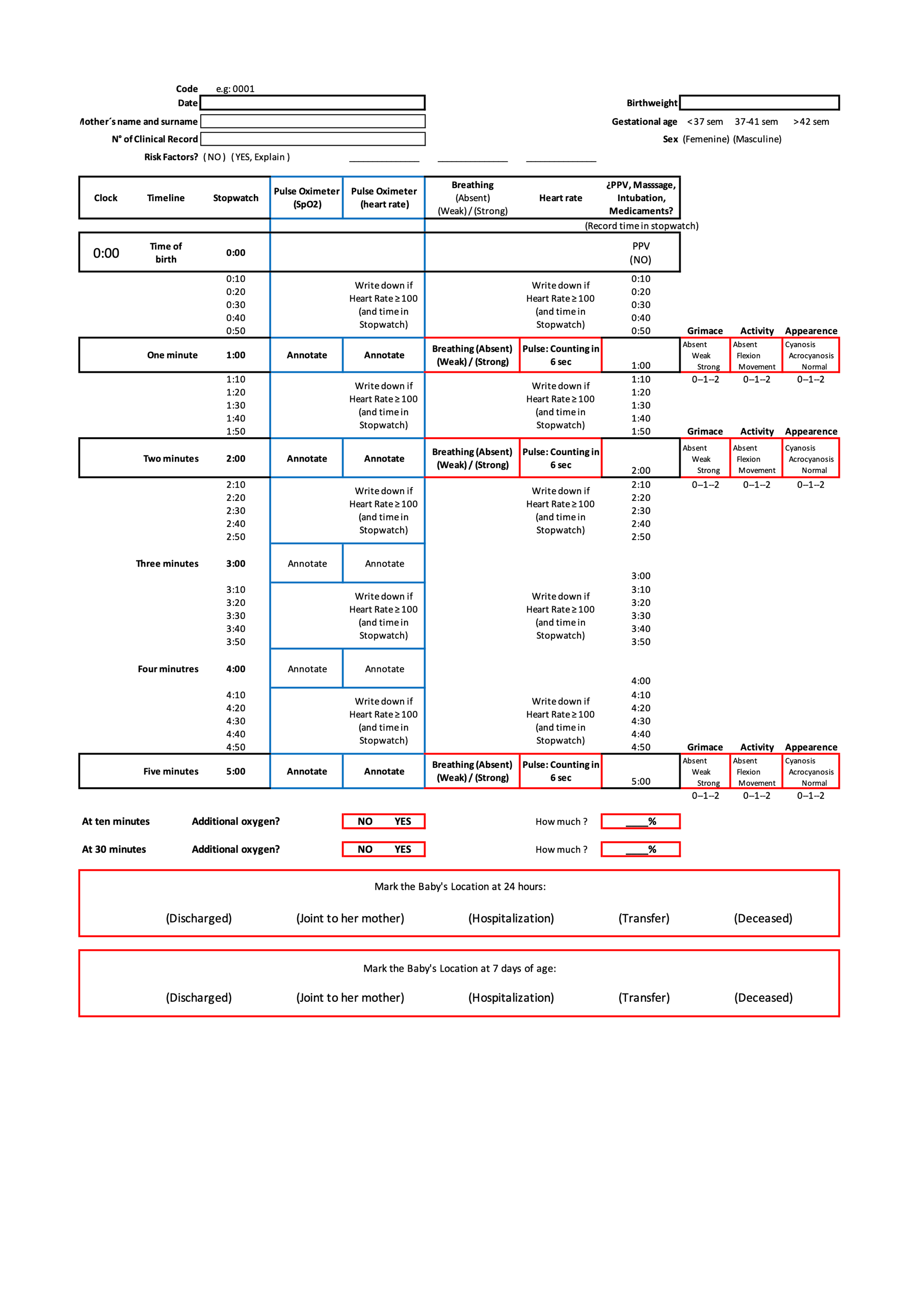
Figure 3s** Delivery room observation sheet
